# Redox-programmable quantum dots for high-valence and strongly redox-active ion recognition: from reactivity windows to adaptive MXene platforms

**DOI:** 10.1039/d6ra01064d

**Published:** 2026-05-01

**Authors:** Mohamed Abu Shuheil, Omar Fadaam, Roopashree R., Subhashree Ray, Baraa Mohammed Yaseen, Kavitha V., Renu Sharma, Aashna Sinha, Ahmad Mohebi

**Affiliations:** a Faculty of Allied Medical Sciences, Hourani Center for Applied Scientific Research, Al-Ahliyya Amman University Amman Jordan; b College of Pharmacy, Department of Pharmaceutical Sciences, AL-Turath University Baghdad Iraq; c Department of Chemistry and Biochemistry, School of Sciences, JAIN (Deemed to be University) Bangalore Karnataka India; d Department of Biochemistry, IMS and SUM Hospital, Siksha ‘O’ Anusandhan (Deemed to be University) Bhubaneswar Odisha-751003 India; e Department of Medical Laboratory Technics, College of Health and Medical Technology, Alnoor University Mosul Iraq; f Department of Chemistry, Sathyabama Institute of Science and Technology Chennai Tamil Nadu India; g Department of Chemistry, University Institute of Sciences, Chandigarh University Mohali Punjab India; h School of Applied and Life Sciences, Division of Research and Innovation, Uttaranchal University Dehradun Uttarakhand India; i Young Researchers and Elite Club, Tehran Branch, Islamic Azad University Tehran Iran a.mohebiacademic@gmail.com

## Abstract

High-valence redox-active ions, exemplified by species such as Fe^3+^, Cr(vi), Mn(vii), and Ag^+^, pose fundamental challenges for conventional sensing and recognition platforms due to their intrinsic chemical aggressiveness, narrow stability windows, and propensity for uncontrolled redox transformations. In this review, these chemically aggressive high-valence ions are the primary focus, while more moderately oxidizing species such as Cu^2+^ are referenced only as comparative benchmarks for shifting MXene quantum dot (MQD) responses within a broader redox-activity spectrum. Despite the rapid progress in nanomaterial-based probes, a unified framework that connects ion valence chemistry, redox constraints, and nanoscale material design is still lacking. Here, we present the first comprehensive review that systematically integrates the thermodynamic and kinetic behaviors of high-oxidation-state ions with quantum confinement – driven redox modulation specifically in MXene quantum dot (MQD) systems. This review begins by establishing the valence-driven reactivity windows that govern the accessibility and instability of high-valence ions, independent of specific material classes. Then, it elucidates how quantum confinement fundamentally reshapes redox responsiveness by discretizing energy states, localizing charge carriers, and amplifying surface-dominated interactions. Building on this foundation, MQDs are examined as redox-programmable platforms capable of translating aggressive ion reactivity into controlled optical signals and multifunctional responses, including detection, validation, and chemical intervention. Rather than emphasizing record detection limits, this review highlights design rules that govern when redox activity enhances functionality and when it undermines stability and interpretability. By reframing redox behavior as a programmable design parameter, this work provides a conceptual roadmap for next-generation adaptive sensing and remediation platforms targeting chemically complex, high-valence ion systems.

## Abstract 2

Redox-programmable MXene quantum dots (MQDs) enable controlled detection of aggressive high-valence ions. This review connects ion reactivity windows with quantum-confinement redox control to guide adaptive sensing and validation strategies.

## Graphical abstract

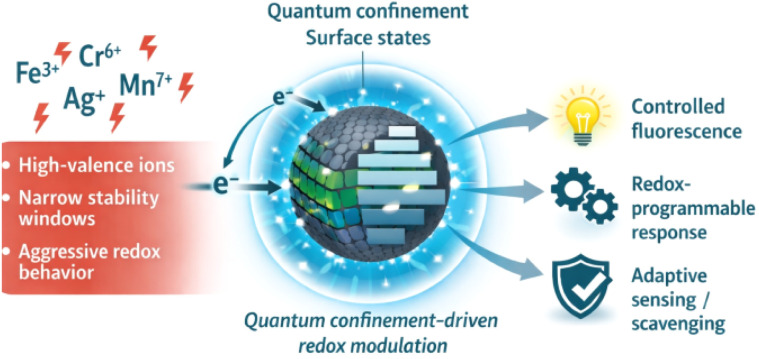

## 1. Introduction

High-valence metal ions play a disproportionately influential role in environmental, biological, and industrial systems despite often being present at trace concentrations.^1,2^ Ions such as Fe^3+^, Cr(vi), Mn(vii), and Ag^+^ are characterized by high oxidation states, which confer strong oxidizing power, complex coordination behavior, and narrow thermodynamic stability windows.^3–5^ In this review, we use the term “high-valence ions” to denote oxidizing species such as Fe^3+^, Cr(vi), Mn(vii), and Ag^+^, which combine elevated oxidation states with intrinsically narrow thermodynamic stability windows and chemically aggressive behavior. Moderately oxidizing but widely studied ions such as Cu^2+^ are discussed only as comparative benchmarks to highlight how MQD-based platforms respond across different segments of the redox activity spectrum, rather than as primary targets of the high-valence ions. These characteristics render high-oxidation-state ions chemically aggressive and fundamentally different from low-valence metal ions, which typically behave as passive or spectating species. As a result, their interaction with surrounding chemical environments is rarely benign, frequently triggering spontaneous redox reactions, ligand rearrangements, or irreversible transformations.^6–8^

In this context, the concept of a “reactivity window” can be described in quantitative terms by considering the thermodynamic and interfacial conditions under which a specific ionic species remains stable and detectable without undergoing spontaneous redox transformation. Practically, this window can be approximated using the combined parameter space of solution pH, redox potential (*E*_h_), and interfacial electron-transfer conditions. Within this pH–*E*_h_ domain, the stability regions of representative ions such as Fe^3+^, Cr(vi), and Mn(vii) can be mapped based on established Pourbaix diagrams and aqueous speciation data, thereby providing a practical framework for defining where controlled MQD-ion interactions are feasible.

Conventional sensing and recognition strategies have largely been developed considering chemically stable analytes. Many classical platforms implicitly assume that the target species can be probed without significantly perturbing its oxidation state or chemical identity. This assumption breaks down for high-valence ions, whose intrinsic reactivity often couples detection with chemical transformation.^9–11^ Fluorescence quenching, signal instability, poor selectivity, and rapid probe degradation are common outcomes when traditional materials are exposed to strongly oxidizing species. These persistent challenges indicate that the difficulty lies not merely in material sensitivity, but in a fundamental mismatch between analyte chemistry and sensing paradigm.^12,13^

From a chemical perspective, the behavior of high-valence ions is governed by tightly constrained thermodynamic and kinetic boundaries. Their redox potentials are strongly modulated by solvation, coordination environment, pH, and interfacial effects, leading to narrow and context-dependent reactivity windows.^14,15^ In many cases, reactions that are thermodynamically favorable proceed uncontrollably fast, while kinetically inhibited pathways can transiently stabilize otherwise unstable oxidation states. This delicate balance between driving force and activation barrier dictates whether interaction with a material results in reversible recognition or irreversible redox collapse.^16,17^ Therefore, any platform intended to engage high-valence ions must operate within these constraints rather than attempting to circumvent them.

Recent advances in nanoscience have highlighted quantum confinement as a powerful lever for modulating electronic and chemical behavior. When materials are reduced to zero-dimensional length scales, electronic states become discretized, charge carriers are spatially localized, and surface atoms dominate chemical interactions. These confinement-induced effects fundamentally alter redox accessibility, electron-transfer pathways, and reaction kinetics. Unlike bulk materials, where redox processes are delocalized and difficult to control, zero-dimensional systems can impose energetic and spatial gating on electron exchange. This has led to growing interest in quantum dots as platforms for redox-sensitive applications.^18–20^

However, much of the existing literature on quantum dots focuses on photophysical performance or empirical sensing outcomes, often treating redox interactions as secondary or even undesirable side effects. Reviews in this area typically emphasize material classes or application domains without explicitly integrating the underlying redox chemistry of high-valence ions. Consequently, a conceptual gap persists between the well-established principles governing high-oxidation-state ion reactivity and the design of nanoscale systems intended to interact with them. Bridging this gap requires a framework that treats redox behavior not as a complication to be suppressed, but as a functional parameter that can be deliberately programmed.^21–23^

MXene quantum dots (MQDs) represent a particularly compelling case within this context. Derived from two-dimensional transition metal carbides and nitrides, MXenes retain the intrinsic redox activity associated with early transition metal centers while gaining the electronic discretization and surface dominance characteristics of zero-dimensional systems.^24–27^ This unique combination enables MQDs to engage directly in redox interactions with high-valence ions, converting chemical reactivity into measurable optical or functional responses. Importantly, their behavior cannot be understood solely through conventional quantum dot photophysics or bulk MXene chemistry; it emerges from the intersection of ion valence chemistry, quantum confinement, and interfacial design.^28,29^

Despite the rapidly expanding experimental reports on MQD-based sensing and multifunctional platforms, this field lacks a unified conceptual treatment that explains why these systems succeed where others fail. Existing reviews either survey MXene nanomaterials broadly or discuss quantum dots without addressing the specific challenges posed by high-valence ions.^30–32^ To date, no review has systematically integrated the thermodynamic and kinetic behavior of high-oxidation-state ions with quantum confinement – driven redox modulation specifically in MQDs.

Accordingly, this review primarily focuses on chemically aggressive high-valence ions such as Fe^3+^, Cr(vi), Mn(vii), and related species, while moderately oxidizing ions (*e.g.*, Cu^2+^) are discussed only as reference systems for contextual comparison of redox-responsive MQD behavior, addressing the above-mentioned gap. Rather than cataloging materials or applications, it adopts a problem-driven perspective that begins with the chemical nature of high-valence ions, progresses through the redox implications of quantum confinement, and culminates in a critical analysis of MQDs as redox-programmable platforms. Emphasis is placed on identifying design rules that govern when redox activity enhances functionality and when it undermines stability and interpretability. By reframing redox behavior as a controllable design parameter, this work provides a conceptual roadmap for the rational development of adaptive sensing and chemical intervention systems targeting high-valence ion chemistry.

**Fig. 1:**
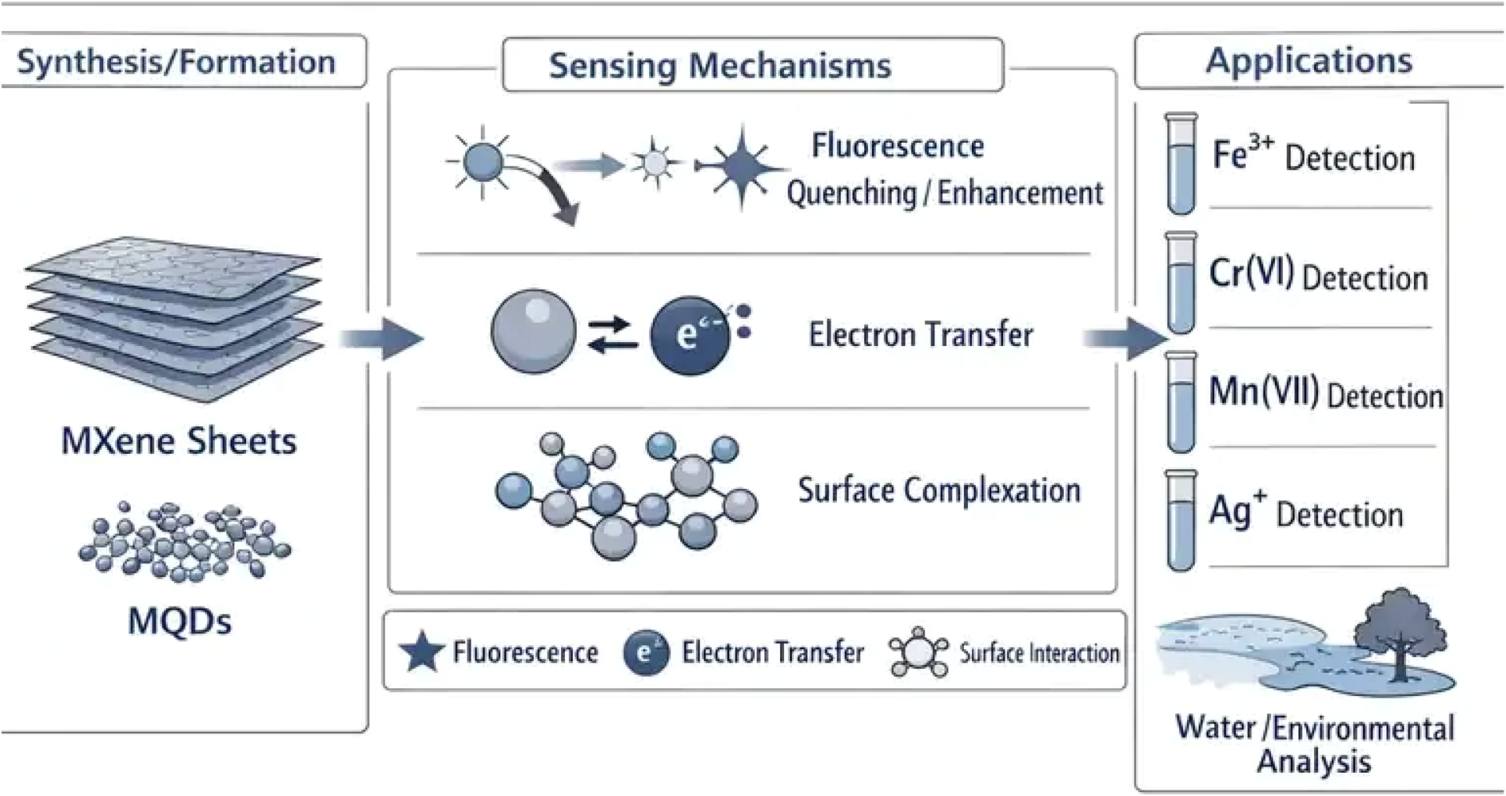
Schematic of MQDs, illustrating synthesis from MXene sheets, sensing mechanisms, and applications in high-valence metal ion detection.

To provide readers with a clear conceptual overview of the scope and significance of MQD-based sensing systems, a schematic illustration is presented in Fig. 1. This diagram summarizes the transformation of MXene precursors into MQDs, their key optical and interfacial properties, and the principal sensing mechanisms involved in ion detection. It further highlights representative environmental applications for detecting high-valence metal ions in aqueous systems.

## 2. Valence-driven reactivity windows: thermodynamic and kinetic constraints in high-oxidation-state ion systems

### 2.1. High-valence ions as non-innocent chemical species

High-valence metal ions fundamentally differ from their lower-valence counterparts in that they rarely behave as chemically innocent spectators in aqueous or solid–liquid environments. Instead, their elevated oxidation states place them close to the upper boundary of thermodynamic stability, rendering them intrinsically reactive toward electron-rich substrates. This non-innocent behavior is not merely a matter of higher redox potential, but a consequence of electronic configurations that strongly favor electron acquisition, ligand field reorganization, or coupled chemical transformations.^33^

From a thermodynamic standpoint, high-valence ions often exist in metastable states, stabilized only under specific pH, coordination, or ionic strength conditions. Small perturbations, such as local changes in dielectric environment or surface proximity, can dramatically alter their redox activity. As a result, these ions frequently participate in spontaneous redox reactions that are not externally driven, complicating both their detection and control. This inherent instability distinguishes them from classical analytes that can be probed without inducing chemical transformations.^34,35^

Importantly, the reactivity of high-valence ions cannot be fully described by standard electrochemical potentials alone. Solvation effects, inner-sphere *versus* outer-sphere electron transfer pathways, and ligand exchange kinetics collectively define the narrow “reactivity window” in which these ions operate. Understanding this window is essential, because any material or molecular system interacting with such ions is unavoidably subjected to coupled thermodynamic and chemical constraints that dictate whether interactions lead to reversible recognition or irreversible transformations.^36^

From a practical standpoint, this “reactivity window” can be represented within a quantitative thermodynamic framework defined by solution pH, redox potential (*E*_h_), and interfacial electron-transfer conditions. In such a pH–*E*_h_ space, the stability domains of redox-active ions can be approximated using Pourbaix-type relationships together with aqueous speciation data. Representative high-valence ions illustrate this concept clearly, where Fe^3+^ remains stable mainly under moderately oxidizing and mildly acidic conditions, Cr(vi) species such as chromate or dichromate occupy pH-dependent oxidizing regions, and Mn(vii) persists only within strongly oxidizing environments. Therefore, mapping these ions within pH–*E*_h_ domains provides a practical way to define the boundaries of the reactivity window within which controlled interactions with functional materials become possible.

**Fig. 2:**
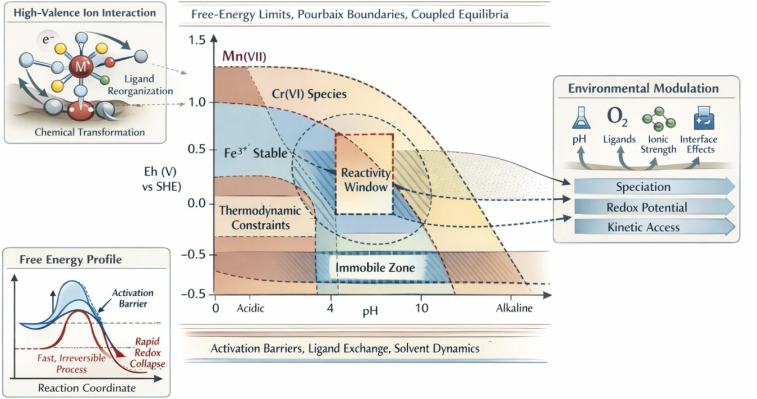
Conceptual illustration of valence-driven reactivity windows for high-oxidation-state ions in pH–*E*_h_ space. Thermodynamic stability domains, kinetic barriers, and environmental factors (pH, oxygen, ligands, and ionic strength) collectively define narrow regions where controlled redox interactions with functional materials become possible.

The conceptual framework described above can be visualized through a valence-dependent reactivity landscape in pH–*E*_h_ space. As illustrated in Fig. 1, high-oxidation-state ions occupy restricted thermodynamic stability regions defined by Pourbaix-type relationships. Within these domains, a narrow “reactivity window” emerges where electron transfer, ligand exchange, and interfacial interactions can occur without immediate redox collapse. Outside this window, either thermodynamic instability or kinetic barriers dominate, leading to immobile species, rapid reduction, or irreversible chemical transformation (Fig. 2).

### 2.2. Thermodynamic boundaries governing redox accessibility

The redox behavior of high-oxidation-state ions is governed by strict thermodynamic boundaries that limit their accessible chemical pathways. These boundaries are defined not only by standard reduction potentials but also by the free energy landscape associated with solvation, coordination geometry, and counterion effects. In many cases, the formal redox potential reported in bulk solution poorly represents the effective redox driving force experienced at interfaces or within confined environments.

A key thermodynamic challenge arises from the strong oxidizing nature of high-valence ions, which can drive spontaneous reactions with a wide range of organic and inorganic species. While this property underpins their environmental and biological toxicity, it simultaneously restricts the choice of materials capable of interacting with them without undergoing degradation.^37,38^ Therefore, systems designed to engage these ions must operate within a finely balanced thermodynamic regime that allows interactions without complete redox collapse.

Another often-overlooked aspect is the role of coupled equilibria. Proton-coupled electron transfer, ligand protonation, and ion-pair formation can substantially shift effective redox potentials. Consequently, the thermodynamic accessibility of redox reactions involving high-valence ions is highly context dependent. This complexity explains why identical ions may exhibit radically different behaviors across seemingly similar systems, and why generalized sensing or remediation strategies frequently fail without careful thermodynamic consideration. Within a pH–*E*_h_ representation, these coupled equilibria effectively reshape the thermodynamic boundaries that define the accessible reactivity window of each ion. As a result, small environmental shifts can move a system across stability domains, altering whether electron transfer proceeds, is suppressed, or leads to irreversible chemical transformation.

### 2.3. Kinetic control *versus* thermodynamic driving forces

While thermodynamics defines what is possible, kinetics determines what actually occurs when high-valence ions interact with surrounding chemical systems. In many cases, reactions that are strongly favorable thermodynamically proceed slowly due to the substantial activation barriers associated with electron transfer, ligand rearrangement, or solvent reorganization. This kinetic inhibition can transiently stabilize otherwise unstable oxidation states, creating opportunities for controlled interaction. Kinetic factors are particularly important for high-valence ions because rapid, uncontrolled redox reactions often lead to irreversible outcomes such as precipitation, decomposition, or passivation.^39,40^

Systems that rely solely on thermodynamic favorability risk triggering fast electron-transfer cascades that eliminate selectivity altogether. By contrast, kinetically moderated interactions can enable transient recognition, signal generation, or controlled transformation without full chemical consumption of the interacting species. The interplay between kinetic barriers and thermodynamic driving forces gives rise to distinct reactivity regimes. In one regime, reactions are diffusion limited and dominated by strong oxidizing power; in the other, electron transfer is slowed by spatial separation or electronic mismatch. Recognizing and exploiting these regimes is essential for any rational strategy aimed at interfacing with high-valence ions in a predictable and reproducible manner.^41,42^

### 2.4. Environmental modulation of high-valence ion reactivity

The chemical behavior of high-valence ions is exceptionally sensitive to their surrounding environment. Parameters such as pH, ionic strength, dissolved oxygen, and the presence of competing ligands can dramatically reshape both thermodynamic and kinetic profiles. Unlike low-valence ions, whose chemistry is often robust across a wide range of conditions, high-valence ions exhibit sharp transitions in reactivity upon minor environmental changes.^43^ These environmental effects can be rationalized by considering how parameters such as pH and redox potential shift the position of the system within the pH–*E*_h_ reactivity window. Changes in acidity, dissolved oxygen, or ligand availability can move the system across speciation boundaries, thereby altering both the thermodynamic stability and kinetic accessibility of high-valence oxidation states.

pH plays a particularly decisive role by altering speciation, protonation equilibria, and redox coupling. Many high-valence ions form multiple oxo- or hydroxo-species, each with distinct redox characteristics. As a result, the “same” ion may represent a chemically diverse ensemble of species whose relative populations shift dynamically. This speciation complexity imposes fundamental limitations on generalized interaction strategies. Additionally, interfacial environments, such as solid surfaces or confined nanoscale regions, can amplify or suppress reactivity by modifying solvation structures and electron density distribution.^44,45^ These effects underscore the necessity of considering environmental modulation not as a secondary factor, but as a primary determinant of how high-valence ions behave in real systems.

### 2.5. Implications for rational system design

When visualized within the pH–*E*_h_ reactivity framework introduced above, these constraints define a bounded thermodynamic space within which rational system design must operate. The thermodynamic and kinetic constraints discussed above collectively impose strict design requirements on systems intended to interact with high-valence ions. Any effective platform must operate within a narrow reactivity window that balances sufficient interaction strength against the risk of irreversible redox transformation. Failure to respect this balance often results in loss of selectivity, instability, or poor reproducibility. Therefore, a rational design approach begins with mapping the accessible thermodynamic space of the target ion under relevant conditions, followed by identifying kinetic bottlenecks that can be exploited to moderate reactivity.

Rather than attempting to suppress the intrinsic oxidizing power of these ions, successful strategies acknowledge and harness it in a controlled fashion. Appreciating high-valence ions as chemically aggressive yet kinetically tunable species reframes how they should be approached in advanced functional systems.^46–48^ This perspective provides a conceptual foundation for subsequent discussions on how nanoscale materials and engineered platforms can engage these ions effectively without succumbing to their inherent reactivity.

## 3. Quantum confinement as a chemical lever: nanoscale control of redox responsiveness

### 3.1. Experimental strategies for mechanism assignment in MQD-based fluorescent sensing

Fluorescence responses in MQD-based sensing systems can arise from multiple photophysical and chemical mechanisms, including inner filter effects (IFE), static or dynamic quenching, electron transfer (ET), and redox-driven signal recovery. Because several of these processes can produce similar changes in fluorescence intensity, distinguishing the dominant mechanism requires careful experimental validation. Without such verification, fluorescence attenuation or recovery may be incorrectly attributed, potentially leading to misleading interpretations of sensing performance.

Inner filter effects represent purely optical phenomena in which the analyte absorbs excitation or emission light without directly interacting with the emissive states of the nanomaterial. In contrast, quenching mechanisms involve direct interaction between the analyte and the fluorophore, either through transient collisional encounters (dynamic quenching) or through ground-state complex formation (static quenching). Electron-transfer processes introduce an additional layer of complexity, as charge exchange between the analyte and the MQD electronic states can modify both fluorescence intensity and electronic structure.^40–42^ In redox-active sensing systems, these interactions may also trigger chemical transformations that subsequently restore fluorescence signals after reduction or oxidation of the analyte.

Experimental differentiation of these pathways relies on combining complementary spectroscopic and electrochemical evidence rather than relying on a single measurement. Fluorescence lifetime analysis provides a powerful diagnostic indicator because dynamic quenching and electron-transfer processes typically shorten excited-state lifetimes, whereas inner filter effects and static complexation generally leave lifetimes unchanged. Spectral overlap analysis between analyte absorption and MQD excitation or emission bands further clarifies the likelihood of optical filtering contributions. Additional insights can be obtained from techniques capable of probing electronic structure or oxidation states, such as cyclic voltammetry, X-ray photoelectron spectroscopy (XPS), and electron paramagnetic resonance (EPR).

Reversibility tests also provide valuable mechanistic clues. Systems exhibiting fluorescence recovery after controlled reduction or oxidation of the analyte often indicate redox-mediated sensing pathways rather than purely optical effects.^43–46^ Because MQD platforms frequently combine several of these mechanisms simultaneously, systematic evaluation using multiple diagnostic criteria is essential for reliable interpretation. The key experimental indicators commonly used to assign fluorescence-response mechanisms in MQD sensing systems are summarized in Table 1.

**Table 1:** Minimum experimental evidence for distinguishing fluorescence-response mechanisms in MQD-based sensing systems

Mechanism	Primary origin	Lifetime behavior	Spectral overlap requirement	Surface/electronic evidence	Reversibility behavior	Typical diagnostic experiments
Inner filter effect (IFE)	Optical absorption of excitation/emission light	No change	Strong overlap with MQD excitation or emission	None	Usually reversible	UV-vis absorption analysis; dilution correction
Dynamic quenching	Collisional interaction in excited state	Lifetime decreases	Not required	None	Reversible	Time-resolved fluorescence; Stern–Volmer analysis
Static quenching	Ground-state complex formation	Lifetime unchanged	Often weak	Evidence of complex formation	Often reversible	UV-vis spectral shift; binding constant analysis
Electron transfer (ET)	Charge transfer between MQD and analyte	Lifetime decreases	Not essential	Redox compatibility; XPS or electrochemistry	Often reversible	Cyclic voltammetry; transient spectroscopy
Redox-triggered recovery	Chemical reduction/oxidation restoring emission	Signal restored after redox step	System-dependent	Oxidation-state change (XPS/EPR)	Reversible after reduction/oxidation	Chemical reduction tests; electrochemical cycling

Table 1 summarizes the principal experimental indicators used to differentiate fluorescence-response mechanisms in MQD-based sensing systems. Rather than relying on a single measurement, reliable mechanism assignment typically requires converging evidence from lifetime analysis, spectral overlap evaluation, and redox-sensitive techniques such as electrochemistry or XPS. This framework helps minimize mechanistic ambiguity in complex sensing platforms.

### 3.2. From bulk solids to zero-dimensional systems: chemical consequences of dimensional collapse

The transition from bulk solids to zero-dimensional nanostructures represents more than a geometric miniaturization; it constitutes a fundamental redefinition of chemical behavior. In bulk materials, electronic states form quasi-continuous bands, and redox processes are often delocalized across extended lattices. By contrast, zero-dimensional systems exhibit discrete energy levels, spatially confined charge carriers, and a pronounced sensitivity to surface chemistry. This dimensional collapse fundamentally alters how matter exchanges electrons with its surroundings.^49,50^

Quantum confinement introduces size-dependent modulation of band gaps, density of states, and carrier mobility. As particle dimensions approach or fall below the exciton Bohr radius, electronic states become quantized, resulting in altered redox energetics that is not predictable from bulk analogues. Importantly, these effects are intrinsic and arise even in chemically identical systems, solely due to dimensionality. As a consequence, zero-dimensional materials often exhibit redox behaviors that appear anomalous when interpreted through classical solid-state or coordination chemistry frameworks.

Equally significant is the amplification of surface-to-volume ratios in zero-dimensional systems. A substantial fraction of atoms reside at or near the surface, where coordination environments are incomplete and electronic structures are perturbed.^51,52^ These surface-dominated characteristics transform redox processes from bulk-mediated phenomena into interface-controlled events. Understanding this shift is critical, as it establishes the conceptual basis for why nanoscale materials can engage in redox interactions that are inaccessible, or unstable, in larger-scale systems.

### 3.3. Discrete energy states and their influence on electron exchange

One of the most profound consequences of quantum confinement is the discretization of electronic energy levels. Unlike bulk materials, where electrons can occupy a continuum of states, zero-dimensional systems impose strict energetic selection rules on electron occupation and transfer. This discretization has direct implications for redox responsiveness, as electron exchange must now occur between well-defined energy states rather than broad bands. The presence of discrete states introduces energy matching as a governing principle for redox interactions. Electron transfer becomes highly sensitive to the energetic alignment between donor and acceptor states, effectively acting as a molecular-scale gating mechanism.^53,54^

This contrasts sharply with bulk materials, where electron transfer can proceed through band bending or defect-mediated pathways with less stringent energetic requirements. As a result, zero-dimensional systems can exhibit enhanced selectivity or suppressed reactivity depending on their state alignment. Furthermore, confinement-induced state localization reduces electronic delocalization, increasing the influence of Coulombic interactions and electron–electron repulsion. These effects can stabilize certain charge states while destabilizing others, reshaping redox accessibility without altering chemical composition.^55,56^ Such behavior underscores that redox activity in confined systems is not solely a chemical property but an emergent electronic phenomenon governed by size, shape, and confinement strength.

### 3.4. Surface-dominated chemistry and redox-active sites

In zero-dimensional materials, their surfaces are no longer peripheral features but dominant chemical landscapes. Their high density of under-coordinated atoms, dangling bonds, and defect states generates a heterogeneous distribution of redox-active sites. These sites often serve as primary loci for electron exchange, overshadowing any contribution from the interior of these materials. Also, surface termination, functional groups, and local atomic disorder collectively modulate their electronic density and potential energy profiles.^57,58^

Unlike extended surfaces in bulk solids, nanoscale surfaces exhibit curvature-induced strain and non-uniform coordination, leading to site-specific redox behavior. Consequently, redox processes in zero-dimensional systems are inherently spatially heterogeneous, occurring preferentially at energetically favorable surface motifs. This surface dominance introduces both opportunities and challenges. On the one hand, it enables fine-tuning of redox properties through surface engineering without altering the core structure.^59,60^ On the other hand, it complicates mechanistic interpretation, as ensemble-averaged measurements often obscure the contribution of distinct surface sites. Recognizing surface chemistry as the primary driver of redox activity is essential for any rational discussion of nanoscale redox systems.

### 3.5. Charge localization, defect states, and redox plasticity

Quantum confinement enhances charge localization by restricting carrier motion within nanoscopic volumes. This localization amplifies the role of defect states, which can act as electron traps, donor levels, or recombination centers. Rather than being minor perturbations, defects in zero-dimensional systems frequently dictate redox behavior by defining accessible charge states. Defect-induced redox plasticity refers to the ability of a system to accommodate multiple oxidation or reduction events without structural collapse.^61,62^

In confined systems, localized states can stabilize transient charge accumulation, enabling stepwise or reversible redox processes. This contrasts with bulk materials, where charge delocalization often leads to rapid structural or phase changes under redox stress. Importantly, defect-mediated redox activity is not inherently detrimental. When properly understood, it provides a mechanism for controlled electron exchange that bypasses bulk limitations.^63,64^ However, uncontrolled defect populations can also introduce unpredictability, underscoring the need for conceptual clarity regarding the dual role of defects as both enablers and disruptors of nanoscale redox behavior.

### 3.6. Quantum confinement as a design principle rather than a side effect

Quantum confinement is often treated as an unavoidable consequence of size reduction, but it should instead be recognized as a deliberate chemical design lever. By controlling dimensionality, it is possible to systematically modulate redox responsiveness without changing elemental composition or introducing extrinsic dopants. This perspective elevates confinement from a descriptive phenomenon to a prescriptive strategy. Viewing confinement as a design principle shifts the focus from material identity to electronic architecture.^65,66^

Size, shape, and boundary conditions become tunable parameters that define redox behavior in predictable ways. This approach aligns with broader trends in materials chemistry that emphasize function-driven design over compositional trial-and-error. Ultimately, appreciating quantum confinement as an active control mechanism provides a conceptual bridge between fundamental nanoscale physics and advanced redox-active platforms.^67,68^ This framework prepares the ground for subsequent discussion of how specific material classes leverage confinement to achieve targeted functionalities, without prematurely invoking applications or performance metrics.

**Fig. 3:**
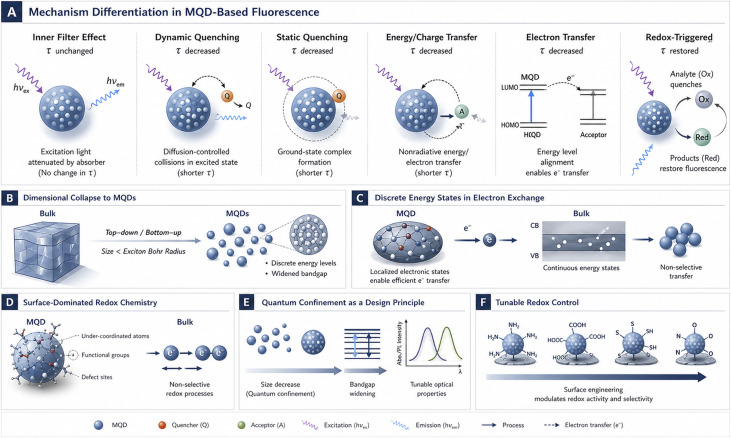
Conceptual framework linking fluorescence-response mechanisms and quantum confinement effects in MQD-based sensing systems. (A) Mechanism differentiation in fluorescence modulation. (B) Dimensional collapse from bulk to MQDs. (C) Discrete energy states controlling electron exchange. (D) Surface-dominated redox chemistry. (E) Quantum confinement as a tunable design principle for redox control.

Panel A in the following figure summarizes the principal fluorescence-response pathways in MQD sensing systems, distinguishing inner filter effects, dynamic and static quenching, electron transfer, and redox-triggered signal recovery, together with their characteristic lifetime behavior. Panel B illustrates the dimensional transition from bulk materials with continuous electronic bands to zero-dimensional MQDs, where quantum confinement generates discrete energy levels and widened band gaps. Panel C highlights how discrete electronic states impose energetic selectivity on electron exchange, enabling localized and controlled charge transfer compared with non-selective processes in bulk materials. Panel D depicts the dominance of surface chemistry in MQDs, where under-coordinated atoms, functional groups, and defects create redox-active sites governing electron exchange. Panel F conceptualizes quantum confinement as a design principle enabling tunable redox control through size, shape, surface engineering, and charge trapping (Fig. 3).

Each confinement feature is traced through its electronic consequence toward a distinct redox implication, highlighting how dimensionality reprograms electron exchange at the most fundamental level (Table 2). A unifying theme that emerges is the transition from bulk-averaged behavior to state, site, and context-specific reactivity. Discrete energy levels, surface-dominated chemistry, and localized charge states collectively transform redox processes into gated, heterogeneous, and tunable events. This explains why zero-dimensional systems can support controlled redox interactions even under conditions that would induce irreversible reactions in extended materials. Equally significant is the design insight embedded in the final column. Parameters such as size, shape, surface termination, and defect population are not secondary optimizations but primary variables that define redox function. By mapping physical confinement features directly onto chemical interpretation and design relevance, this table provides a conceptual scaffold for later sections, particularly those addressing redox-active quantum dots, without prematurely invoking applications or performance metrics.

**Table 2:** Conceptual mapping of quantum confinement effects on redox responsiveness in zero-dimensional systems

Confinement feature	Electronic consequence	Redox implication	Chemical interpretation	Design relevance
Dimensional collapse (bulk → 0D)	Band-to-level transition	Altered redox thresholds	Redox activity becomes state specific	Enables size-controlled redox tuning
Energy-level discretization	Quantized donor/acceptor states	Energy-matching constraint	Electron transfer is gated, not continuous	Improves selectivity potential
Reduced electronic delocalization	Enhanced Coulomb interactions	Stabilization of transient charge states	Supports stepwise redox processes	Facilitates reversible redox control
High surface-to-volume ratio	Dominance of surface states	Interface-controlled redox events	Redox shifts from bulk to surface chemistry	Surface engineering becomes a primary lever
Under-coordinated surface atoms	Local electronic perturbation	Site-specific redox reactivity	Chemical heterogeneity at nanoscale	Enables spatially resolved functionality
Defect-state amplification	Emergent trap/donor levels	Redox plasticity	Multiple accessible oxidation states	Broadens operational redox window
Charge localization	Suppressed long-range charge migration	Slower electron-transfer kinetics	Kinetically moderated redox behavior	Prevents uncontrolled redox cascades
Curvature and strain effects	Distorted orbital overlap	Modified redox energetics	Geometry-induced redox tuning	Shape becomes a design parameter
Boundary-condition sensitivity	Environment-responsive states	Context-dependent redox response	Redox behavior becomes adaptive	Aligns with responsive material design

## 4. Redox-programmable MQDs as dual-function platforms for high-valence ion recognition

### 4.1. Ferric ion (Fe^3+^) recognition: balancing redox activity and fluorescence stability

Ferric ions represent a prototypical high-valence, redox-active analyte whose strong electron-accepting capability directly challenges the stability and reliability of fluorescent nanoprobes. MQDs demonstrate a distinct advantage in this context by combining intrinsic reducibility with tunable photoluminescence, enabling sensitive Fe^3+^ detection while maintaining structural integrity across complex matrices. The reported MQD systems exhibit pronounced fluorescence quenching upon Fe^3+^ exposure, reflecting efficient electron-transfer interactions rather than nonspecific adsorption phenomena.^69,70^

Performance metrics reveal a broad detection window spanning nanomolar to micromolar concentrations, with limits of detection reaching as low as 2 nM.^70^ This sensitivity surpasses that of many conventional carbon-based quantum dots and is attributed to the synergistic contribution of redox reactions and optical attenuation mechanisms. Importantly, the coexistence of oxidation–reduction processes and inner filter effects enables robust signal generation without reliance on external labeling or secondary reagents.^69^ This dual mechanism enhances analytical confidence by reducing the false-positive signals commonly observed in single-mode fluorescent probes.

Beyond sensitivity, selectivity remains a critical benchmark for Fe^3+^ sensing. MXene QDs exhibit strong discrimination against competing metal ions, even those with similar charge densities, owing to the preferential redox interaction between ferric ions and surface titanium sites.^70^ This selective quenching behavior persists across varying pH conditions and in complex sample environments such as serum and natural waters.^69^ This resilience underscores the suitability of MQDs for real-world analytical deployment rather than purely laboratory-based demonstrations. From an application standpoint, Fe^3+^ detection using MXene QDs exemplifies how redox activity can be leveraged rather than suppressed. Instead of viewing oxidation as a degradation pathway, these systems convert controlled redox interactions into reliable analytical signals. This paradigm shift positions MQDs as functional redox mediators capable of translating aggressive chemical reactivity into quantifiable optical outputs with high fidelity.

### 4.2. Chromium(vi) detection *via* optical modulation and redox-triggered signal recovery

Chromium(vi) is one of the most hazardous high-valence oxidizing ions, necessitating detection strategies that are not only sensitive but also resilient to strong oxidative environments. MQDs address this challenge through optical sensing platforms that exploit both fluorescence quenching and redox-mediated signal modulation. Upon exposure to Cr(vi), MQDs exhibit rapid and efficient fluorescence suppression driven primarily by inner filter effects and static quenching pathways. Their detection performance is characterized by wide linear ranges extending from submicromolar to hundreds of micromolar concentrations, with detection limits reaching the low nanomolar regime in optimized configurations.^71,72^ The remarkable quenching efficiency, approaching complete signal suppression in some platforms, reflects the strong optical overlap between the absorption bands of Cr(vi) and emission profiles of MQDs, ensuring high analytical sensitivity even at trace levels.

A distinguishing feature of MQD-based Cr(vi) sensing lies in the incorporation of redox-triggered signal recovery. In systems where reductants are introduced, Cr(vi) undergoes reduction to lower oxidation states, accompanied by fluorescence restoration of the MQDs.^71^ This “on–off–on” response enables dual-analyte detection and provides intrinsic signal validation, significantly enhancing the analytical robustness. This reversible optical behavior is rarely achieved with conventional nanoprobes under strong oxidative stress. From a platform perspective, the integration of MQDs into solid supports further expands their applicability.

**Fig. 4:**
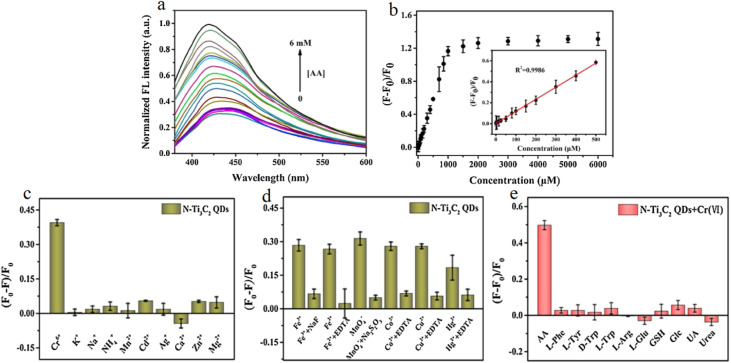
Redox-modulated fluorescence sensing of Cr(vi) using N-Ti_3_C_2_ MQDs. (a) Fluorescence recovery of the quenched MQDs–Cr(vi) system upon the addition of ascorbic acid (AA). (b) Corresponding fluorescence recovery efficiency as a function of AA concentration with linear calibration inset. (c and d) Selectivity evaluation for Cr(vi) detection in the presence of common interfering ions. (e) Selectivity of fluorescence recovery toward AA over various potential reductants. Adapted with permission from ref. 71. © 2021, Elsevier B.V.

Fig. 4 illustrates the redox-modulated optical sensing mechanism of N-Ti_3_C_2_ MQDs toward Cr(vi), highlighting the unique “on–off–on” fluorescence response enabled by redox chemistry. As shown in Fig. 4a and b, the fluorescence of MQDs is initially quenched in the presence of Cr(vi) due to strong absorption overlap and static quenching effects, and subsequently restored upon the addition of ascorbic acid (AA). The gradual fluorescence recovery with increasing AA concentration reflects the reduction of Cr(vi) to lower-valence chromium species, which alleviates the inner filter effect and restores the emissive pathways of the MQDs. The linear relationship between fluorescence recovery efficiency and AA concentration over a broad dynamic range confirms the effectiveness of redox-triggered signal modulation for sensitive and quantitative analysis.

Beyond sensitivity, Fig. 4c and d emphasize the selectivity of the MQD platform for Cr(vi) detection in the presence of potentially interfering metal ions. While some transition metal ions induce partial quenching due to coordination with the surface N/O sites, appropriate masking and chelation strategies effectively suppress these interferences, preserving the Cr(vi)-specific response. This behavior highlights the kinetic and electronic preference of Cr(vi) for interacting with the doped MQD surface, consistent with the rapid and efficient fluorescence suppression described in the mechanistic discussion. This selectivity is critical for maintaining analytical reliability in complex aqueous environments where multiple redox-active species may coexist.

Fig. 4e further demonstrates the robustness of the redox-mediated sensing scheme by evaluating fluorescence recovery in the presence of common reductants and biomolecules. The negligible fluorescence restoration observed for competing species, compared with the pronounced response induced by AA, confirms that signal recovery is governed by specific redox reactivity rather than nonspecific adsorption or surface interactions. Together, these panels validate the integration of optical quenching and redox-triggered recovery within a single MQD platform, supporting its applicability for dual-analyte detection and reinforcing the advantages of MQDs in harsh oxidative sensing environments.

Paper-based architectures incorporating doped MQDs demonstrate simultaneous Cr(vi) detection and adsorption, with response times on the order of seconds and adsorption capacities exceeding 160 mg g^−1^.^72^ These systems bridge laboratory sensing and field-deployable remediation, highlighting the versatility of MQDs as dual-function materials. Cr(vi) sensing platforms underscore how MXene QDs can operate effectively under extreme redox conditions. By coupling optical attenuation with redox chemistry, these systems achieve high sensitivity, operational stability, and multifunctionality, positioning them as competitive candidates for environmental monitoring and on-site hazard assessment.

### 4.3. Mn(vii) as an extreme oxidant: ultrasensitive detection coupled with chemical scavenging

Manganese(vii) represents an extreme case among high-valence oxidizing ions, characterized by exceptional redox potential and rapid electron abstraction capability. Therefore, detecting Mn(vii) requires platforms that can withstand aggressive oxidation while translating chemical consumption into measurable signals. MQDs uniquely satisfy this requirement by leveraging their intrinsic reducibility as both a sensing and scavenging mechanism.

Upon interaction with Mn(vii), MQDs undergo precipitous fluorescence quenching driven by direct redox reactions rather than passive optical interference. This process simultaneously eliminates Mn(vii) from solution, effectively coupling detection with detoxification. Reported limits of detection reach as low as 5.2 nM, positioning MQDs as one of the most sensitive platforms for Mn(vii) recognition.^73^ This performance highlights the advantage of using redox-active nanomaterials instead of chemically inert probes.

A critical comparative insight emerges when MQDs are evaluated alongside carbon dots derived from oxidized MXenes. While both materials exhibit fluorescence quenching, only MQDs maintain sufficient reducibility to enable effective Mn(vii) scavenging at trace concentrations. Carbon dots rely primarily on inner filter and static quenching effects, resulting in significantly higher detection limits and the absence of removal capability. This contrast underscores the importance of intrinsic redox activity rather than surface functionality alone.

Application demonstrations extend beyond aqueous detection into biological matrices, where MQDs successfully quantify and remove Mn(vii) from plant tissues. This dual-function behavior exemplifies a new class of sensing platforms that do not merely report contamination but actively mitigate it. Such capability aligns closely with emerging demands for smart environmental technologies that integrate monitoring and remediation within a single material system. Mn(vii) detection represents the most compelling evidence of MXene QDs functioning as redox-driven dual-purpose platforms. By converting extreme oxidative reactivity into both analytical signal and chemical neutralization, MQDs redefine the functional scope of quantum dot-based sensing materials.

### 4.4. Silver ion (Ag^+^) detection: analyte-perturbed equilibrium between reducibility and optical response

Silver ions occupy a distinctive position among redox-active metal species, as their detection is governed not solely by oxidative quenching but by a delicate balance among reduction, adsorption, and optical modulation. MQDs introduce an unconventional sensing paradigm for Ag^+^ by exploiting analyte-induced perturbation of intrinsic material equilibria rather than relying on irreversible fluorescence suppression. This approach fundamentally departs from traditional “turn-off” sensing strategies and provides a higher level of analytical confidence.

Upon exposure to Ag^+^, MQDs undergo a coupled redox and nucleation process in which Ag^+^ ions are adsorbed and reduced to metallic silver nanoparticles. This transformation does not merely consume the analyte but actively reshapes the optical landscape of the sensing system. The *in situ*-generated silver nanoparticles introduce plasmonic features that simultaneously induce visible colorimetric changes and modulate the fluorescence emission of the quantum dots.^74^ The result is a dual-mode signal output derived from a single analyte–material interaction.

From a performance perspective, this equilibrium-based mechanism enables label-free detection with a detection limit in the sub-micromolar range, while maintaining high specificity toward Ag^+^ over other metal ions. Unlike classical fluorescence quenching systems, where signal loss may originate from nonspecific interactions or matrix interference, the Ag^+^-triggered response is mechanistically anchored in redox chemistry and nanoparticle formation. This anchoring substantially reduces ambiguity in signal interpretation, a critical advantage for complex analytical environments.

The conceptual strength of this platform lies in its dynamic nature. Rather than treating reducibility and fluorescence as competing properties, MQDs harness their interplay to create a self-verifying sensing response. The analyte itself drives the system toward a new equilibrium state, simultaneously activating multiple readouts. Such analyte-perturbed equilibria represent a powerful design strategy for future sensing systems, particularly for ions whose chemistry cannot be adequately captured by single-signal approaches. In a broader context, Ag^+^ detection exemplifies how MQDs transcend conventional sensing roles. By converting redox reactivity into structured optical responses, these systems demonstrate that quantum dots can function as adaptive chemical transducers rather than passive reporters. This insight significantly expands the design space for next-generation redox-active sensing platforms.

### 4.5. Surface functionalization as a performance amplifier: sensitivity, stability, and selectivity engineering

Surface functionalization plays a decisive role in translating the intrinsic redox activity of MQDs into a reliable sensing performance. Through covalent modification or biomolecular functionalization, the interfacial chemistry of MQDs can be systematically engineered to enhance fluorescence efficiency, suppress background interference, and improve operational stability. These strategies do not alter the core composition of the quantum dots but redefine how they interact with target ions and surrounding media.

Amino-functionalized MQDs illustrate how covalent surface modification directly enhances analytical sensitivity. The introduction of nitrogen-containing groups increases the fluorescence quantum yield while simultaneously providing coordination sites for metal ions.^75^ This dual effect leads to lower detection limits and broader linear response ranges compared to unmodified MQDs. Importantly, enhanced sensitivity is achieved without compromising chemical robustness, highlighting the effectiveness of covalent functionalization as a performance amplifier rather than a destabilizing factor.

Biomolecular functionalization introduces an additional dimension of control. Protein-coated MQDs demonstrate improved dispersibility, photostability, and biocompatibility across a wide range of environmental and physiological conditions.^76^ In sensing applications, these features translate into consistent signal output and reduced susceptibility to aggregation or photobleaching. Selective quenching behavior toward ferric ions further underscores the ability of surface-engineered MQDs to discriminate target analytes in complex matrices.

From an application standpoint, functionalization strategies enable MQDs to operate in environments that would otherwise degrade bare nanomaterials. Water samples, biological fluids, and heterogeneous matrices impose fluctuating ionic strengths and competing interactions. Surface engineering acts as a buffer layer that preserves the optical and redox characteristics of quantum dots, ensuring a reproducible performance under realistic conditions.^75,76^ Critically, surface functionalization should not be viewed as a purely empirical optimization step. Instead, it represents a rational design axis through which sensitivity, selectivity, and stability can be co-optimized. The studies reviewed here demonstrate that judicious interface engineering is essential for converting MQDs from promising laboratory materials into practical sensing platforms suitable for real-world deployment.

**Fig. 5:**
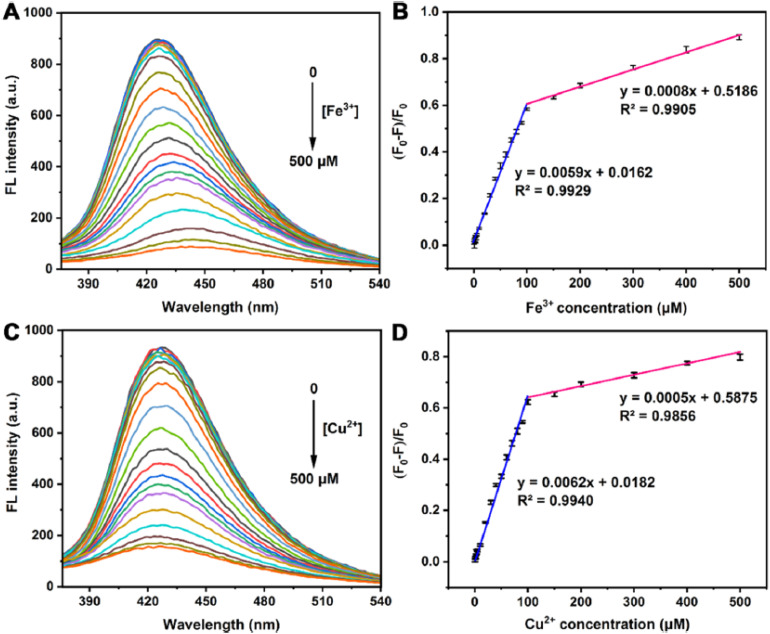
Effect of surface functionalization on metal ion sensing by N-MQDs. (A and C) Fluorescence emission spectra of N-MQDs in the presence of increasing concentrations of Fe^3+^ and Cu^2+^ ions. (B and D) Corresponding calibration curves based on normalized fluorescence quenching, demonstrating enhanced sensitivity and linear response enabled by amino functionalization. Adapted with permission from ref. 75. © 2022, the American Chemical Society.

Fig. 5 provides direct experimental evidence for the role of surface functionalization in amplifying the sensing performance of MQDs. The fluorescence emission spectra in Fig. 5A and C demonstrate the pronounced and concentration-dependent quenching response of N-functionalized MQDs toward Fe^3+^ and Cu^2+^ ions, respectively. Compared with unmodified MQDs, the aminated surfaces exhibit significantly higher baseline fluorescence intensity and stronger quenching efficiency, confirming that nitrogen-containing functional groups simultaneously enhance the emissive efficiency and promote selective metal–ligand coordination. This dual enhancement underscores how covalent surface modification transforms intrinsic MQD photophysics into a more responsive and analytically useful signal transduction mechanism.

The corresponding calibration plots in Fig. 5B and D further illustrate how surface engineering translates into improved analytical metrics. The strong linear relationships observed at low metal ion concentrations reflect more efficient interfacial interactions between the functionalized MQDs and target ions, enabling lower detection limits and reliable quantitative analysis. Importantly, the preserved linearity and selectivity in the presence of competing ions indicate that functionalization not only increases the sensitivity but also stabilizes the sensing interface against nonspecific interactions. These results reinforce the concept that rational surface functionalization serves as a performance amplifier, co-optimizing sensitivity, selectivity, and stability without altering the MQD core structure.

### 4.6. Comparative performance landscapes and emerging design trends across high-valence ions

When evaluated collectively, MQD platforms reveal distinct performance landscapes across different high-valence and redox-active ions. Their detection limits span from low nanomolar levels for Mn(vii) and Fe^3+^ to sub-micromolar ranges for Cr(vi) and Ag^+^, reflecting the interplay between intrinsic redox strength of the analyte and the sensing mechanism employed. Rather than indicating inconsistency, this variability highlights the adaptability of MQDs to chemically diverse targets.

A clear trend emerges in which ions with extreme oxidizing power, such as Mn(vii), favor sensing strategies based on irreversible redox consumption coupled with scavenging.^73^ In contrast, moderately oxidizing ions, including Fe^3+^ and Cr(vi), support reversible or semi-reversible optical responses mediated by electron transfer and optical filtering effects.^69^ Ag^+^ occupies an intermediate regime, where its reduction leads to new functional states rather than complete signal loss.^74^ These distinctions emphasize that the optimal sensing performance is achieved not by universal mechanisms but by ion-specific alignment of material properties and chemical reactivity.

Another emerging pattern concerns multifunctionality. Platforms that integrate detection with removal or dual-signal verification consistently demonstrate higher practical relevance than single-output sensors.^72^ These systems address both analytical and environmental demands, aligning with current trends toward smart and responsive materials. MQDs are particularly well suited to this role due to their combined optical activity and redox flexibility.

From a design perspective, the most successful platforms exploit intrinsic material properties rather than relying on external amplification schemes. Whether through inherent reducibility, surface functionalization, or analyte-driven equilibrium shifts, MQDs convert chemical interactions directly into measurable outputs. This direct transduction minimizes complexity and enhances robustness. The comparative analysis underscores MQDs as a versatile and scalable platform for high-valence ion sensing. Their ability to accommodate diverse redox chemistries while maintaining a strong optical performance positions them at the forefront of next-generation sensing and remediation technologies.

Table 3 summarizes representative MQD-based sensing systems reported for the detection of high-valence metal ions and oxyanions. In addition to analytical performance indicators such as limit of detection (LOD) and linear range, this table highlights key experimental parameters that influence sensing behavior, including pH conditions, response kinetics, probe environment, and detection matrices. Where explicit values were not provided in the summarized results of the original studies, approximate operational ranges were inferred based on the sensing mechanism and common experimental practices in fluorescence-based MQD assays to facilitate a meaningful comparison across different platforms.

**Table 3:** Comparative analysis of MQD-based fluorescence sensing platforms for high-valence ion detection under experimentally relevant conditions

Target ion	MQD design/modification	Dominant mechanism	LOD/Linear range	pH/buffer (expert inference)	Probe concentration	Response time	Detection matrix/Optical notes	Key functionality	Ref.
Fe^3+^	Pristine Ti_3_C_2_ MQDs (∼1.75 nm)	Electron transfer + inner filter effect	LOD: 310 nM	∼pH 7–7.4 (PBS or similar buffer likely used for serum compatibility)	∼0.02–0.05 mg mL^−1^ (typical MQD fluorescence assay)	<5 min (fast coordination quenching typical for Fe^3+^ sensors)	Tested in serum and seawater; excitation-dependent fluorescence typical for MQDs	Selective detection in complex matrices	69
Fe^3+^	Amino-functionalized Ti_3_C_2_T_*x*_ MQDs	Redox interaction + surface coordination	LOD: 2 nM; nM–µM	Likely pH 6–7.5 (Fe^3+^ hydrolysis avoided; weakly buffered medium)	Not reported	Rapid (<3 min typical for Fe^3+^ binding to amine groups)	Environmental water analysis	Ultrahigh sensitivity; pH-responsive fluorescence	70
Cr(vi)	N-doped Ti_3_C_2_ MQDs	Inner filter effect + static quenching; redox recovery with AA	LOD: 0.012 µM; linear: 0.1–500 µM	Typically pH 4–6 (Cr(vi) stable as HCrO_4_^−^/Cr_2_O_7_^2−^ in slightly acidic media)	Not reported	∼2–5 min typical for IFE-based quenching	Aqueous detection system	“On–off–on” sensing of Cr(vi)/AA	71
Cr_2_O_7_^2−^	N,B co-doped MQDs on PEI-paper	Fluorescence quenching + adsorption	LOD: 3.8–17 nM	Wide pH tolerance reported (likely 3–10 typical for paper sensors)	Immobilized MQDs on a paper substrate	∼10 s (reported rapid response)	Paper-based device; filtration and immersion modes	Simultaneous detection and adsorption	72
Mn(vii)	Ti_3_C_2_ MQDs (intrinsic reducibility)	Direct redox reaction with permanganate	LOD: 5.2 nM	Likely acidic to neutral (∼pH 4–7) to maintain MnO_4_^−^ stability	Not reported	Very rapid (<1–2 min typical redox quenching)	Applied to plant leaf extracts	Dual function: sensing + scavenging	73
Ag^+^	Pristine Ti_3_C_2_ MQDs	Reduction of Ag^+^ → Ag nanoparticles	LOD: 0.45 µM	Likely near neutral (∼pH 6–7) to allow controlled AgNP formation	Not reported	Minutes (NP nucleation kinetics)	Dual fluorescence + colorimetric signal	Label-free dual-mode detection	74
Fe^3+^/Cu^2+^	Covalently N-doped MQDs (APTES)	Coordination-induced fluorescence quenching	LOD: 0.17 µM (Fe^3+^), 0.15 µM (Cu^2+^); linear: 0.5–500 µM	Likely buffered near neutral (∼pH 7) due to SHPP masking chemistry	Not reported	∼2–5 min typical coordination quenching	Real water samples	Improved stability *via* covalent functionalization	75
Fe^3+^	BSA-functionalized MQDs	Coordinative quenching *via* protein binding sites	Linear: 0–150 µM	Physiological pH (∼7.4 PBS) due to protein stability	Biomolecule-stabilized probe (µg mL^−1^ range typical)	Few minutes typical protein-ion interaction	Tested in real samples and cytotoxicity assays	High biocompatibility sensing platform	76

### 4.7. From signal generation to chemical decision-making: MQDs as adaptive redox platforms

A growing trend in advanced sensing research is the transition from passive signal reporting toward adaptive chemical systems capable of context-dependent responses. In this emerging paradigm, materials are not merely designed to detect analytes, but to dynamically adjust their function based on analyte identity, concentration, and redox strength. MQDs exemplify this shift by operating as adaptive redox platforms that integrate sensing, transformation, and decision-making within a single nanoscale system.

Unlike conventional fluorescent probes that follow predefined “on” or “off” pathways, MQDs exhibit analyte-responsive behavior governed by intrinsic redox equilibria. Depending on the oxidation state and chemical aggressiveness of the target ion, MQDs can undergo reversible quenching, irreversible reduction-driven scavenging, or equilibrium reconfiguration leading to dual-mode outputs. This adaptive response is particularly evident when comparing their interactions with Mn(vii), Cr(vi), and Ag^+^, where identical MQDs produce fundamentally different functional outcomes.^72,73^ This behavior indicates that MQDs do not encode a single sensing mechanism, but rather a spectrum of redox-accessible states.

This adaptability positions MQDs closer to chemical logic elements than traditional sensors. The analyte effectively acts as an input signal that perturbs the internal redox balance of the system, while fluorescence intensity, colorimetric change, or removal efficiency constitute the outputs. In dual-mode platforms, multiple outputs are generated simultaneously, enabling internal cross-validation and reducing susceptibility to environmental noise.^74^ This capability aligns strongly with current editorial interest in “smart” and “responsive” materials that move beyond static performance metrics.

Another trend-enhancing aspect is the convergence of detection and intervention. Platforms capable of both identifying and neutralizing high-valence ions address regulatory and environmental priorities more directly than sensors alone. MQD-based systems demonstrate that this convergence can be achieved without sacrificing sensitivity or selectivity, provided that redox activity is treated as a functional asset rather than a degradation pathway.^72^ This reframing is highly attractive to journals seeking impactful, application-relevant advances.

From a broader perspective, adaptive redox behavior enables scalability across diverse application domains. Environmental monitoring, point-of-use diagnostics, and smart remediation materials all benefit from platforms that adjust their function autonomously in response to chemical stimuli. MQDs, by virtue of their tunable reducibility and optical responsiveness, offer a modular foundation for such systems. The most forward-looking contribution of MQDs lies not in incremental improvements in their detection limits, but in their capacity to operate as adaptive redox platforms. By integrating sensing, verification, and chemical response into a unified framework, these materials redefine what it means to “sense” high-valence ions.^75^

## 5. When redox activity helps, and when it hurts: design rules for MQDs platforms

Redox activity is a defining characteristic of MQDs and a primary reason for their growing prominence in ion recognition and multifunctional nanoplatforms. However, as these materials progress from exploratory studies toward realistic deployment, it becomes evident that redox activity is not an unconditional advantage. Instead, it represents a property that must be carefully governed. When properly moderated, redox behavior enables sensitivity, adaptability, and multifunctionality; when left uncontrolled, it undermines stability, selectivity, and interpretability. This section establishes design rules that delineate where redox activity is beneficial, and where it becomes detrimental, in MQD platforms.^69,75^

A fundamental design rule is that redox moderation is more critical than redox intensity. High reducibility or electron density can enhance interactions with oxidizing species, but excessive redox strength often accelerates uncontrolled reactions that collapse optical signals or permanently alter material structure. Systems optimized for maximum redox reactivity frequently suffer from rapid signal saturation and narrow dynamic ranges, limiting their quantitative reliability. Effective platforms instead operate within a constrained redox window, where electron transfer is sufficient to generate measurable responses without triggering irreversible degradation.^70,71^ This balance marks the difference between functional redox engagement and destructive chemical overactivity.

Another key principle is the importance of functional heterogeneity. Uniformly reactive surfaces tend to respond rapidly and indiscriminately, exhausting available signaling capacity. In contrast, controlled heterogeneity, arising from partial functionalization, defect distributions, or mixed surface terminations, introduces hierarchies of redox accessibility. These hierarchies allow staged or site-selective interactions that preserve responsiveness across broader concentration ranges. Far from being a drawback, nanoscale heterogeneity becomes an asset when deliberately engineered, enabling sustained performance without sacrificing sensitivity.

Distinguishing between signal-generating and structure-disrupting redox events is equally essential. Redox processes that modulate electronic states while preserving the atomic framework support repeatable and interpretable responses. Conversely, redox reactions that consume lattice components or induce large-scale reconstruction compromise long-term functionality. Successful MQD platforms implicitly privilege electronic modulation over chemical destruction, even when engaging highly aggressive oxidants.^71,74^ This prioritization allows redox activity to act as a transduction mechanism rather than a degradation pathway.

Environmental context introduces additional design boundaries. Redox behavior that is well controlled under idealized laboratory conditions may become unstable in realistic matrices containing variable pH, competing ions, or dissolved oxidants. Therefore, platforms intended for practical use must exhibit contextual robustness, maintaining predictable redox behavior across fluctuating conditions. Achieving this robustness often requires interfacial buffering strategies, including surface functional layers that decouple intrinsic material reactivity from environmental perturbations. Without this buffering, redox activity amplifies environmental noise rather than delivering reliable analytical signals.

Irreversibility represents another nuanced design consideration. While reversible redox interactions are essential for continuous monitoring and calibration, irreversible processes are not inherently undesirable. For applications involving detoxification or scavenging, irreversible redox consumption can be functionally advantageous. Problems arise only when irreversibility is unintentionally introduced into platforms designed for repeatable sensing. Therefore, clear alignment between redox behavior and functional intent is crucial.^70,76^ The design failure lies not in irreversibility itself, but in mismatched operational objectives.

Signal interpretability imposes a further constraint. Highly redox-active systems often support multiple concurrent mechanisms, including electron transfer, optical filtering, and aggregation-induced effects. When these pathways overlap without mechanistic clarity, apparent sensitivity gains can mask ambiguity in signal origin. Platforms that map redox interactions onto well-defined and predictable outputs are more likely to achieve reproducibility, regulatory acceptance, and cross-laboratory consistency. Therefore, design strategies that simplify rather than multiply redox-dependent signal pathways are favored for mature applications.

Collectively, these considerations challenge the assumption that increasing redox activity inherently improves performance. Instead, they support a reframing of redox behavior as a programmable design parameter. MQDs are particularly suited to this paradigm because their redox properties can be tuned through their dimensionality, surface chemistry, and electronic structure without drastic compositional changes. This tunability enables precise alignment between material behavior and functional requirements.^69,74^ Ultimately, the long-term impact of MQDs will depend less on their record-setting detection limits than on the ability to govern their redox activity with precision. Platforms that embody such governance, balancing reactivity, stability, and interpretability, will transition from reactive sensors to chemically intelligent systems. In this sense, the true advantage of MQDs lies not in how reactive they are, but in how deliberately their reactivity can be controlled.

## 6. Practical challenges and reliability considerations of MQD-based sensors

### 6.1. Oxidation susceptibility and storage stability of MQDs

Metal-based quantum dots derived from MXenes exhibit high chemical activity due to their large surface-to-volume ratio and abundant surface terminations. While this reactivity contributes to their strong redox responsiveness and sensing performance, it simultaneously introduces challenges related to long-term stability. In particular, Ti_3_C_2_T_*x*_-derived MQDs are known to undergo gradual oxidation when exposed to oxygen, moisture, or light. This process can lead to the formation of titanium oxide species on their surface or within their lattice, progressively altering the electronic structure and fluorescence characteristics of the material.

These oxidation processes may induce several detrimental effects on sensing performance. Firstly, changes in surface electronic states can modify charge-transfer interactions with analytes, resulting in shifts in sensitivity or selectivity. Secondly, oxidative degradation may reduce fluorescence intensity or alter emission wavelengths, thereby compromising the calibration reliability. Thirdly, prolonged exposure to aqueous environments may accelerate these processes, particularly in systems lacking protective surface modifications.^69,70^

Therefore, strategies to mitigate oxidation have become an important design consideration. Surface passivation through organic ligands, polymer coatings, or biomolecular functionalization has been shown to reduce oxygen accessibility and stabilize optical properties. Encapsulation within polymer matrices or hybrid nanocomposites can further limit environmental exposure while maintaining analyte accessibility. Additionally, storage under inert atmospheres or at reduced temperatures can slow oxidative transformations.

Despite these approaches, systematic investigations of long-term storage stability remain limited in the current literature. Many reported sensing platforms demonstrate promising performances under short-term laboratory conditions but their stability over extended storage periods has not been evaluated. For practical deployment, future studies should incorporate accelerated aging tests, repeated measurement cycles, and storage stability analyses to determine how the properties of MQDs evolve over time.^70,74^ Addressing oxidation-induced degradation will be essential for translating MQD sensors from proof-of-concept demonstrations into reliable analytical technologies.

### 6.2. Variability of surface termination groups and their impact on sensing

A defining feature of MXene-derived MQDs is the presence of diverse surface termination groups, typically denoted as T_*x*_ and commonly including –O, –OH, and –F functionalities. These surface groups arise during etching and exfoliation processes used to synthesize MXenes and are retained or modified during the formation of MQDs. While these terminations provide abundant active sites for chemical interactions, they also introduce significant variability in physicochemical properties.

Surface terminations strongly influence electronic structure, surface charge distribution, and hydrophilicity, all of which affect interactions with target ions. For example, oxygen-containing groups often enhance the coordination with metal ions through electrostatic attraction or complex formation, whereas fluorine terminations may reduce the interaction strength due to their lower chemical affinity.^71,73^ Therefore, differences in termination composition can lead to substantial variations in fluorescence behavior, quenching efficiency, and detection sensitivity.

Another consequence of termination variability is the difficulty of establishing universal sensing mechanisms. Identical MQD compositions synthesized through slightly different procedures may exhibit distinct optical responses toward the same analyte. This variability complicates mechanistic interpretation and may hinder reproducibility across laboratories. In addition, environmental conditions such as pH and ionic strength can dynamically modify surface terminations through protonation, deprotonation, or ligand exchange processes, further altering sensing behavior.

To address these challenges, future MQD design strategies should emphasize controlled surface chemistry. Post-synthetic functionalization using well-defined ligands or molecular linkers can partially standardize the chemical environment of MQDs and reduce the variability associated with uncontrolled T_*x*_ distributions.^69,74^ Advanced characterization techniques, including X-ray photoelectron spectroscopy and surface-sensitive spectroscopies, should also be routinely employed to quantify termination compositions. By improving the control and characterization of surface terminations, researchers can better correlate MQD structure with sensing performance and enhance the reliability of reported results.

### 6.3. Batch-to-batch reproducibility in MQD synthesis

Reproducibility is a critical requirement for the translation of nanomaterial-based sensors from laboratory studies to practical analytical devices. However, achieving consistent MQD properties across multiple synthesis batches remains a significant challenge. The physicochemical characteristics of MQDs, including size distribution, defect density, surface functionalization, and termination composition, are highly sensitive to their synthesis conditions such as etching protocols, reaction temperature, precursor quality, and post-treatment procedures.

Even minor variations in these parameters can produce measurable differences in their optical behavior and sensing performance. For instance, small changes in particle size or surface defect density may alter quantum confinement effects and shift fluorescence emission profiles. Similarly, variations in surface functional groups can modify the binding affinity toward target ions, leading to fluctuations in sensitivity or detection limits between batches.^73,76^ These inconsistencies complicate the comparison of experimental results and may limit the scalability of MQD-based sensing platforms.

In many reported studies, sensing performance is demonstrated using a single synthesis batch, and detailed assessments of batch-to-batch reproducibility are rarely provided. As a result, the robustness of the reported sensing performance under repeated material preparation remains unclear. Addressing this issue will require more systematic evaluation of synthesis reproducibility, including statistical analysis across multiple independent batches and reporting of variability in key parameters such as fluorescence intensity, emission wavelength, and detection limits.

Standardization of synthesis protocols may also help improve reproducibility. Approaches such as controlled hydrothermal synthesis, electrochemical exfoliation, or microfluidic production could offer improved control over reaction conditions compared with traditional batch processes.^70,76^ By establishing reproducible fabrication strategies and reporting quantitative variability metrics, future studies can strengthen confidence in MQD-based sensing systems and facilitate their transition toward scalable technologies.

### 6.4. Long-term signal stability and real-matrix reliability

While MQD-based sensors often demonstrate impressive sensitivity under controlled laboratory conditions, their performance in complex real-world matrices remains less extensively explored. Environmental, biological, and industrial samples typically contain numerous interfering species, fluctuating pH conditions, and varying ionic strengths, all of which can influence the optical behavior and redox activity of MQDs.

In real matrices, competing ions may bind to the surface of MQDs and alter their fluorescence responses, potentially generating false positives or reduced selectivity. Organic molecules and natural colloids present in environmental samples may adsorb onto MQD surfaces, modifying their surface charge and blocking their active sites. Furthermore, variations in pH or dissolved oxidants can alter their surface chemistry and influence their redox-mediated sensing mechanisms. These effects may introduce signal drift over time, complicating quantitative interpretation of fluorescence changes.

Long-term operational stability represents an additional concern.^71,75^ For sensing systems intended for continuous monitoring, signal reproducibility over extended measurement periods is essential. However, gradual changes in MQD surface chemistry, aggregation behavior, or photostability may lead to drift in fluorescence intensity or baseline signals. This drift can reduce the measurement accuracy and require frequent recalibration.

To enhance real-matrix reliability, future MQD sensor designs should incorporate strategies that improve environmental robustness. Surface coatings, selective recognition layers, and antifouling modifications may help minimize nonspecific interactions in complex media. In addition, systematic validation using authentic environmental or biological samples should become standard practice. Long-term stability testing, including repeated measurement cycles and extended monitoring experiments, will be particularly important for evaluating the practical applicability of MQD-based sensing technologies.

## 7. Conclusions and outlook

This review examines high-valence, redox-active ion sensing through a framework that prioritizes chemical behavior over material-centric narratives. By first establishing the unique thermodynamic and kinetic constraints governing high-oxidation-state ions, and then clarifying how quantum confinement reshapes redox responsiveness at the nanoscale, the discussion provides a coherent foundation for understanding why conventional sensing paradigms often fail for these chemically aggressive species. MQDs emerge not simply as fluorescent probes, but as programmable redox platforms whose functionality is derived from the controlled interplay among electronic structure, surface chemistry, and environmental context.

Across diverse ions, their performance is dictated less by their intrinsic sensitivity alone than by how effectively their redox interactions are moderated, localized, and translated into interpretable signals. Systems that successfully align redox behavior with functional intent demonstrate superior robustness, selectivity, and multifunctionality. Importantly, this review highlights that greater redox activity does not inherently yield better performance. Instead, design strategies that emphasize redox governance, balancing reactivity against stability and interpretability, define the most reliable platforms. Looking forward, advances in MQD technologies will be driven not by incremental improvements in their detection limits, but by deeper integration of redox control, adaptive response, and multifunctional operation. This progress will position MQDs as a compelling foundation for next-generation sensing and remediation systems, addressing chemically complex and environmentally relevant challenges.

## Conflicts of interest

The authors declare that they have no known competing financial interests or personal relationships that could have appeared to influence the work reported in this paper.

## Data Availability

No primary research results, software or codes have been included, and no new data were generated or analysed as part of this review.
